# Denosumab might prevent periprosthetic bone loss after total hip and knee arthroplasties: a review

**DOI:** 10.1186/s42836-021-00068-6

**Published:** 2021-04-12

**Authors:** Jianda Xu, Huan Li, Yuxing Qu, Chong Zheng, Bin Wang, Pengfei Shen, Zikang Xie, Kang Wei, Yan Wang, Jianning Zhao

**Affiliations:** 1 Department of Orthopaedics, Changzhou Traditional Chinese Medical Hospital, Affiliated to Nanjing University of Traditional Chinese Medicine, 25 North Heping Road, Changzhou, 213000 Jiangsu China; 2 Department of Arthroplasty, The First People’s Hospital of Changzhou, Changzhou, 213003 China; 3 Department of Orthopaedics, Jinling Hospital, Nanjing, 210002 Jiangsu China

**Keywords:** Denosumab, Periprosthetic bone loss, Total hip/knee arthroplasty

## Abstract

Total hip arthroplasty and total knee arthroplasty are extensively used for the treatment of the end-stage degenerative joint diseases. Currently, periprosthetic bone loss is still the major cause of aseptic loosening, resulting in implant failures. Previous literature introduced some widely accepted protocols for the prevention and treatment of periprosthetic bone loss, but no guideline has been proposed. Denosumab, a human monoclonal immunoglobulin G2 (IgG2) antibody, can inhibit bone resorption by binding to the receptor activator of nuclear factor kappa-B ligand (RANKL). This article reviews the present findings and evidence concerning the effect of denosumab on the periprosthetic bone loss after total hip arthroplasty and total knee arthroplasty. Overall, the current evidence suggests that denosumab is a promising agent for the treatment of periprosthetic bone loss.

## Introduction

Both total hip arthroplasty (THA) and total kneearthroplasty (TKA) are common orthopaedic procedures for the treatment of the end-stage degenerative joint diseases. Currently, periprosthetic bone loss (PBL) remains the major cause of aseptic loosening that results in implant failures [1]. Previous literature reported some widely accepted protocols for preventing and treating PBL, but no guideline has been proposed [2–6].

Denosumab (Prolia^®^), a human monoclonal immunoglobulin G2 (IgG2) antibody, can inhibit bone resorption by binding to the receptoractivator of nuclear factor kappa-B ligand (RANKL). It was initially engineered by Amgen and used for treating osteoporosis to increase bone mineral density (BMD). Denosumab increases bone mineral density by near-maximally reducing bone resorption [7].

PBL usually starts in the first postoperative year, and gradually slow down after 7 years [2, 3]. To achieve a long-term survival of the implants, multiple medicines have been developed to treat PBL [4]. Bisphosphonates act on osteoclasts and inhibit periprosthetic bone resorption around the implant, but the effect on the local BMD is transient [5]. Alendronate can be used to attenuate the postoperative periprosthetic BMD, but its protective effect on bone density occurs after 6 months, missing the key period of bone resorption secondary to stress shielding happening 3 months after surgery [6].

This article reviews the current findings and evidence regarding the effect of denosumab on the PBL after THA and TKA.

### Why denosumab in THA and TKA?

The receptor activator of nuclear factor κB (RANK) and its ligand (RANKL) signaling system play a critical role in the regulation of bone metabolism. It stimulates osteoclast formation and improves cell function and survival [8]. New osteoclasts are continuously recruited when the prosthetic loosening starts to worsen. Aspenberg *et al.* [9] suggested that targeting osteoclast recruitment via the RANKL system is a potential alternative for preventing periprosthetic loosening. Anti-RANKL agent exerts an effect comparable to high bisphosphonate doses in improving mechanical fixation of screws and fracture healing of cancellous bone [10]. The inhibiting process leads to a decreased bone resorption and increased bone density.

Denosumab, a human monoclonal immunoglobulin G2 (IgG2) antibody, selectively binds to RANKL. It works the same way as osteoprotegerin. Osteoprotegerin is an endogenous product of osteoblasts, and acts as a ‘decoy receptor’ of RANKL and modulates osteoclastic bone resorption [11]. The binding of denosumab with RANKL prevents the RANK from activation on the surface of osteoclasts and their precursors [12], thereby inhibiting bone resorption. Denosumab reduces bone resorption by 86% on average, which is higher than that of other anti-resorptive drugs [13].

Denosumab was initially engineered by Amgen. It is used to treat osteoporosis by increasing the BMD. After a recommended subcutaneous dosage (once every 6 months), the maximum serum concentration of denosumab is often achieved in a median of 10 days. Thereafter, the concentration slowly declines over a period of 3 to 5 months. Pharmacokinetically, it works in a non-linear and dose-dependent fashion [14]. Denosumab, when subcutaneously given, can improve BMD more effectively than bisphosphonate in patients with postmenopausal osteoporosis [15]. It rapidly increases the BMD and strength of osteoporotic cortical bones [16]. In addition, denosumab reduces the risk of fractures in postmenopausal women with osteoporosis. Compared with the placebo, it reduces the risk of hip fractures by 40% over a period of 3 years [13].

### Therapeutic efficacy of denosumab

Periprosthetic bone remodelingdue to stress shielding is a biomechanical behavior post arthroplasty, especially 6 to 12 months after surgery. After THA, the periprosthetic BMD of the proximal femur drops from15% to 40% in elderly patients with osteoporosis [17]. Denosumab can prevent PBL after THA. Especially in Gruen zone 7, the BMD can be increased by as much as 7% (Table 1) [18]. Aro *et al*. (Table 1) [19] suggested that denosumab increases the BMD of proximal femur. It should be administered for 6 to 12 months after surgery, which allows the bone to respond locally to prevent stem migration. Nagoya *et al.* [18] believed that the post-TKA administration of denosumab could significantly prevent early PBL, and reduce periprosthetic tibial bone atrophy for up to 12 months. Murahashi *et al*. (Table 1) [20] confirmed that denosumab helped to attain a better early stability and prognosis by significantly reducing the PBL in the early postoperative period. Ledin *et al*. (Table 1) [21] found that denosumab reduced the early migration of the tibial component by minimizing the total point motion. It provided a better early and long-term stability of the implant.

**Table 1:** The studies included

Serial no.	Author	Denosumab			Control			Comments
		*n*	Age (y)	Methods	*n*	Age (y)	Methods	
1	Nagoya *et al*. [18]	10	78.4 ± 4.3	0.5 μg vitamin D3 daily and 60 mg denosumab every 6 months (1 to 2 weeks and 6 months postoperatively)	10	80.8 ± 2.2	0.75 μg active vitamin D3 daily by oral administration	Denosumab had a definitive effect on the restoration of proximal periprosthetic bone loss, especially in zone 7, after cementless THA. Denosumab contributed to the restoration of decreased periprosthetic BMD to normal levels.
2	Aro *et al*. [19]	33	69.1 ± 5.2	A 60 mg denosumab every 6 months; the first subcutaneous dose of denosumab 1 month before surgery and the second injection at 6 months, the efficacy lasted for 1 year.	32	69.1 ± 5.9	Vitamin D and calcium supplementation was started during the screening visit at a minimum of 2 weeks before administration. The first subcutaneous dose of placebo was given 1 month before surgery and the second injection at 6 months for an effective treatment period of 1 year.	Denosumab increased periprosthetic BMD in the clinically relevant regions of the proximal femur, but the treatment response was not associated with any reduction of initial stem migration.
3	MUrahashi *et al.* [20]	13	76.9 ± 7.3	0.5 μg vitamin D3 daily and 60 mg denosumab every 6 months (the day after surgery, 6 months and 12 months postoperatively)	15	75.3 ± 8.7	0.5 μg active vitamin D3 daily by oral administration	Denosumab treatment significantly reduced periprosthetic BMD loss, even at the early stages after TKA. This therapeutic strategy might facilitate early stable fixation of the prosthesis, which, in turn, might help to prevent early implant migration and reduce the need for revision surgery.
4	Håkan *et al*. [21]	25	66 ± 6.3	1 mL (60 mg) denosumab was given on the first postoperative day after knee replacement surgery and again after 6 months.	25	64 ± 5.5	1 mL saline was given on the first postoperative day after knee replacement surgery and again after 6 months.	Denosumab reduced early migration in total knee replacement and might be beneficial for long-term results.
5	Andreas *et* *al*. [22]	32	58 ± 5	The first subcutaneous injection containing 1 mL (60 mg) of denosumab was given after baseline periprosthetic BMD assessment with dual-energy X-ray absorptiometry scans had been performed and fasting morning blood samples drawn 1 to 3 day(s) postoperatively. The second and final injections were administered 6 months postoperatively.	32	59 ± 5	The first subcutaneous injection containing 1 mL of saline was given after baseline periprosthetic BMD assessment with dual-energy X-ray absorptiometry scans had been performed and fasting morning blood samples drawn 1 to 3 day(s) postoperatively. The second and final injections were administered 6 months postoperatively.	Denosumab potently prevented early periprosthetic bone loss after uncemented THA.

### Potentials and unknowns

#### Tolerability and safety

A proven and well-established regime (60 mg, once every 6 months) is well tolerated up to 10 years in postmenopausal women, even those with renal impairment [23]. BMD improvement still exists and no evidence of plateau is found even used after 10 years. No other denosumab-containing medications are used at the same time. The abdomen and upper arm or thigh are good places for subcutaneous injection. Coskun [24] analyzed the current data and concluded that denosumab was an effective therapeutic option to increase BMD and decrease the level of bone-turnover marker in patients with glucocorticoid-induced osteoporosis. No serious adverse event was reported. The denosumab is degraded into peptides and amino acids through hepatic, rather than renal, metabolism. The anti-fracture efficacy and dose are not significantly impacted by renal impairment [25].

Adverse events include injection site reaction and infection, hypocalcemia, cardiovascular issues, cancer, osteonecrosis of the jaw, *etc*. Among them, hypocalcaemia is a contraindication, but adequate intake of calcium and vitamin D can decrease the transient hypocalcemic effect, especially in patients with the conditions predisposing them to hypocalcemia. Although the RANKL is regularly expressed in some T lymphocytes, no immune dysfunction is found after denosumab therapy [7]. So far, cardiovascular and aortic calcification and glucose tolerance were reported, but further pharmaco-epidemiological studies are warranted to evaluate its long-term efficacy and safety [26, 27].

#### Duration and administration

No consensus has been reached regarding the optimal duration of continuous denosumab therapy. However, the regular assessment of potential risks and benefits is needed. A world-wide, randomized, placebo-controlled trial involving 7868 women with postmenopausal osteoporosis showed that denosumab (60 mg, twice yearly for 3 years) significantly reduced the risks of vertebral, non-vertebral, and hip fractures [13].

Denosumab discontinuation may bring a reversal effects on bone turnover and BMD. The anti-resorptive effects disappear quickly after the treatment withdrawal. Nyström *et al*. (Table 1) [22] conducted a prospective randomized controlled trial. They found that denosumab prevented the early bone resorption around the implants. However, they also found that the biochemical markers of bone metabolism decreased in the first year and rebounded after 2 years. The retardation effect of bone loss lasted only when treatment persisted (Table 1). The authors concluded that denosumab persistently preserves the periprosthetic BMD after uncemented THA. The rebound effect of bone metabolism markers and the loss of gained BMD were also found in the postmenopausal women who suspended denosumab 3 days to 6 months after surgery [28].

A potential risk of multiple vertebral fractures does exist. And patients are not advised to delay or omit denosumab doses due to the relatively poor prognosis of vertebroplasty. The conclusions are based on the arbitrary cut-off of 2 years [29]. Considering the rebound fractures, the European Menopause and Andropause Society recommends that other anti-resorptive drugs be used after denosumab discontinuation, but the protocol is not supported by solid evidence [30].

#### Combination or sequential administration

In patients with postmenopausal osteoporosis, switching to denosumab was associated with higher BMD in the spine and hip than those who continued alendronate after 12 months [31]. The same phenomenon was also found with use of ibandronate [32] and zoledronic acid [15]. Moreover, denosumab increases BMD with the anabolic agent teriparatide in women with postmenopausal osteoporosis [33]. An anabolic agent combined with bone resorption inhibitor may be a promising approach. However, whether it will translate to a higher reduction of fracture risk needs further pharmacological and clinical studies in the future.

## Conclusions

Currently, the overall evidence suggests that denosumab is promising in treating PBL. Sufficiently-sized and long-term randomized controlled trials are needed to find the optimal administration routes and potential side effects in THA and TKA arthroplasties.

## Data Availability

Data sharing not applicable to this review as no datasets were generated or analyzed during this review.

## Abbreviations

THA/TKA: Total hip/knee arthroplasty
IgG2: Immunoglobulin G2
RANKL: Receptor activator of nuclear factor kappa-B ligand
BMD: Bone mineral density
OPG: Osteoprotegerin
RANK: Receptor activator of NF-κB
RANKL: Receptor activator of nuclear factor κB ligand
